# The kisspeptin-GnRH pathway in human reproductive health and disease

**DOI:** 10.1093/humupd/dmu009

**Published:** 2014-03-09

**Authors:** Karolina Skorupskaite, Jyothis T George, Richard A Anderson

**Affiliations:** 1 MRC Centre for Reproductive Health, The Queen's Medical Research Institute, University of Edinburgh, 47 Little France Crescent, Edinburgh EH16 4TJ, UK; 2 Diabetes Trials Unit, Oxford Centre for Diabetes, Endocrinology and Metabolism, University of Oxford, Churchill Hospital, Headington, Oxford OX3 7LJ, UK

**Keywords:** kisspeptin, kisspeptin-neurokinin B-dynorphin, GnRH, LH pulsatility

## Abstract

**BACKGROUND:**

The discovery of kisspeptin as key central regulator of GnRH secretion has led to a new level of understanding of the neuroendocrine regulation of human reproduction. The related discovery of the kisspeptin-neurokinin B-dynorphin (KNDy) pathway in the last decade has further strengthened our understanding of the modulation of GnRH secretion by endocrine, metabolic and environmental inputs. In this review, we summarize current understanding of the physiological roles of these novel neuropeptides, and discuss the clinical relevance of these discoveries and their potential translational applications.

**METHODS:**

A systematic literature search was performed using PUBMED for all English language articles up to January 2014. In addition, the reference lists of all relevant original research articles and reviews were examined. This review focuses mainly on published human studies but also draws on relevant animal data.

**RESULTS:**

Kisspeptin is a principal regulator of the secretion of gonadotrophins, and through this key role it is critical for the onset of puberty, the regulation of sex steroid-mediated feedback and the control of adult fertility. Although there is some sexual dimorphism, both neuroanatomically and functionally, these functions are apparent in both men and women. Kisspeptin acts upstream of GnRH and, following paracrine stimulatory and inhibitory inputs from neurokinin B and dynorphin (KNDy neuropeptides), signals directly to GnRH neurones to control pulsatile GnRH release. When administered to humans in different isoforms, routes and doses, kisspeptin robustly stimulates LH secretion and LH pulse frequency. Manipulation of the KNDy system is currently the focus of translational research with the possibility of future clinical application to regulate LH pulsatility, increasing gonadal sex steroid secretion in reproductive disorders characterized by decreased LH pulsatility, including hypothalamic amenorrhoea and hypogonadotropic hypogonadism. Conversely there may be scope to reduce the activity of the KNDy system to reduce LH secretion where hypersecretion of LH adds to the phenotype, such as in polycystic ovary syndrome.

**CONCLUSIONS:**

Kisspeptin is a recently discovered neuromodulator that controls GnRH secretion mediating endocrine and metabolic inputs to the regulation of human reproduction. Manipulation of kisspeptin signalling has the potential for novel therapies in patients with pathologically low or high LH pulsatility.

## Introduction

Since its discovery, hypothalamic secretion of GnRH has been robustly established as the key pathway that initiates and controls reproductive function. Whilst the pivotal central role played by GnRH remains undisputed, a number of functional limitations of the GnRH neuronal network have been identified. For example, in rats, GnRH neurones lack estrogen receptor (ER)-alpha (Herbison and Theodosis, 1992), the principal ER, thus suggesting the need for an intermediary signalling pathway mediating gonadal feedback.

It was only a decade ago that the discovery of the obligate role of kisspeptin in human puberty revolutionized current understanding of the neuroendocrine regulation of human reproduction (de Roux *et al.*, 2003; Seminara *et al.*, 2003). Kisspeptin, a hypothalamic peptide coded by the KiSS1 gene, is a novel neuromodulator that acts upstream of GnRH, and is sensitive to sex steroid feedback and metabolic cues. Kisspeptin is now recognized as a crucial regulator of the onset of puberty, the regulation of sex hormone-mediated secretion of gonadotrophins, and the control of fertility (Pinilla *et al.*, 2012). The related discovery of a reproductive role for neurokinin B has stimulated further interest in the field. The same functional neuronal network secretes kisspeptin and neurokinin B—now called kisspeptin-neurokinin B-dynorphin (KNDy) neurones as they also co-secrete dynorphin, a well-established opioid inhibitor (Goodman *et al.*, 2007). Exogenous kisspeptin has been administered to healthy volunteers and a limited number of patients, with a view to restoring reproductive function in certain conditions.

In this review, we summarize current understanding of the physiological regulation of GnRH pulse frequency by kisspeptin, and appraise the clinical relevance of the discoveries of kisspeptin and neurokinin B. The focus will predominantly be on human findings, using animal data where human studies are lacking but where there is direct translational potential.

## Methods

A systematic literature search was performed using PUBMED for all English language articles published up to January 2014 using the terms ‘kisspeptin’ and ‘reproduction’. The search was performed without limitations by species although subsequent priority was given to human studies, where available. The initial search identified 390 manuscripts, which were used as background material for the review. In addition, the reference lists of all relevant original research articles and reviews were examined and selected if judged to be relevant. Relevant abstracts from recent scientific meetings were included in the review.

## Discovery of the kisspeptin and KNDy neuronal network

### KISS1 gene, peptide and its receptor

*KISS1*, the gene encoding kisspeptins, was originally identified in 1996 as a suppressor of metastasis in human malignant melanoma (Lee *et al.*, 1996). As it was discovered in Hershey (PA, USA), the gene was named after the famous chocolate ‘Kisses’ produced in the town. The SS in *KiSS1* acknowledges that it is a ‘suppressor sequence’.

The *KISS1* gene is localized to chromosome 1q32 and has four exons, the first two of which are not translated (West *et al.*, 1998). The gene encodes the precursor 145 amino acid peptide, which is cleaved to a 54 amino acid protein (West *et al.*, 1998). To acknowledge its metastasis inhibitory properties, the 54 amino acid transcript was named ‘metastin’ (Ohtaki *et al.*, 2001). This can be further cleaved to 14, 13 and 10 amino acid peptides. The 54 amino acid and the shorter peptides belong to the RF amide group of peptides, sharing the C-terminal sequence of Arg-Phe-NH_2_, and are now collectively referred to as kisspeptins (Kotani *et al.*, 2001).

In 2001, kisspeptin was identified as a ligand for the orphan G-protein coupled receptor 54 (GPR54), which was first described in the rat brain and subsequently in human (then named AXOR12 and hOT7T175) (Lee *et al.*, 1999; Muir *et al.*, 2001; Ohtaki *et al.*, 2001), now termed KISS1R (Gottsch *et al.*, 2009). *KISS1R* maps to chromosome 19p13.3 and includes five exons, encoding a 398 amino acid protein with seven hydrophobic trans-membrane domains (Muir *et al.*, 2001). It has an amino acid sequence close to that of the galanin receptor family (40% identity), although it does not bind either galanin or galanin-like peptide (Lee *et al.*, 1999). Upon binding by kisspeptin, KISS1R activates phospholipase C and recruits secondary intracellular messengers, inositol triphosphate and diacylglycerol, which in turn mediate calcium release and protein kinase C activation to mediate kisspeptin's function (Muir *et al.*, 2001; Liu *et al.*, 2008; Constantin *et al.*, 2009). Activation of Kiss1r results in a biphasic increase in intracellular calcium, with a rapid increase followed by a more sustained second phase (Min *et al.*, 2014). To maintain this second phase and therefore sustain signalling, kisspeptin receptor trafficking involving internalization, recycling and recruitment from an intracellular pool, is required (Min *et al.*, 2014). Without receptor trafficking, the kisspeptin receptor undergoes desensitization following an initial acute phase (Min *et al.*, 2014). Since the discovery of kisspeptin-KISS1R signalling, many different terms have been used to describe its components. The nomenclature used in this review for kisspeptin and its receptor will be that recently recommended by Gottsch *et al.* (2009).

### Functional neuroanatomy of kisspeptin signalling

**Figure 1:**
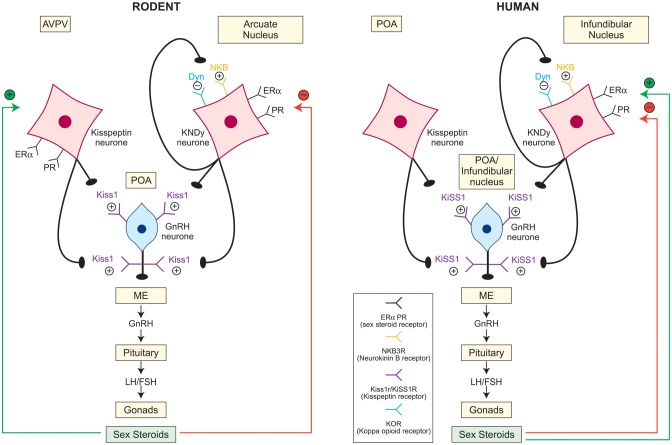
Schematic diagram showing the neuroanatomy of the kisspeptin-GnRH pathway and the relationship between KNDy neurones and GnRH neurones in humans and rodents. Kisspeptin signals directly to the GnRH neurones, which express kisspeptin receptor. The location of kisspeptin neurone populations within the hypothalamus is species specific, residing within the anteroventral periventricular nucleus (AVPV) and the arcuate nucleus in rodents, and within the preoptic area (POA) and the infundibular nucleus in humans. Kisspeptin neurones in the infundibular (humans)/arcuate (rodents) nucleus co-express neurokinin B and dynorphin (KNDy neurones), which via neurokinin B receptor and kappa opioid peptide receptor autosynaptically regulate pulsatile kisspeptin secretion, with neurokinin B being stimulatory and dynorphin inhibitory. Negative (red) and positive (green) sex steroid feedback is mediated via distinct kisspeptin populations in rodents, via the AVPV and the arcuate nucleus, respectively. In humans KNDy neurones in the infundibular nucleus relay both negative (red) and positive (green) feedback. The role of the POA kisspeptin population in mediating sex steroid feedback in humans is incompletely explored. ME, median eminence; +, stimulatory; −, inhibitory; ERα, estrogen receptor alpha; PR, progesterone receptor; Kiss1/KiSS1, kisspeptin; NKB, neurokinin B; Dyn, dynorphin.

GnRH neurones extend from the preoptic area through to the infundibular nucleus (homologue to the arcuate nucleus in other species) of the hypothalamus in humans, whereas in rodents GnRH neurones reside predominantly in the preoptic area (Lehman *et al.*, 1986; Schwanzel-Fukuda and Pfaff, 1989; Clifton and Steiner, 2009) (Fig. 1). GnRH axons project from these nuclei to the median eminence, where GnRH is secreted into the portal circulation in a coordinated and pulsatile manner. Similarly, kisspeptin neurones are located in the rostral preoptic area and the infundibular nucleus in the human hypothalamus (Rometo *et al.*, 2007; Hrabovszky *et al.*, 2010). The anatomical distribution of kisspeptin neurones and their appositions with other hypothalamic endocrine networks are described below. Areas of incongruity between data from human studies and those carried out in other species are also highlighted.

#### Kisspeptin neurone localization in humans

Initial studies of the neuroanatomical distribution of kisspeptin neurones in the human brain carried out in autopsy samples from premenopausal and post-menopausal women localized *KISS1* expression to the infundibular nucleus only (Rometo *et al.*, 2007). A more recent study, using both male and female autopsy samples, has confirmed the localization of the majority of kisspeptin cell bodies in the infundibular nucleus, but identified a second dense population of kisspeptin cells in the rostral preoptic area (Hrabovszky *et al.*, 2010).

Whilst kisspeptin neurones are located in the infundibular/arcuate nucleus across all species, including humans, the rostral population is species specific (Clarkson and Herbison, 2006; Pompolo *et al.*, 2006; Ramaswamy *et al.*, 2008; Clarkson *et al.*, 2009; Hrabovszky *et al.*, 2010). In rodents, the rostral population is located in the anteroventral periventricular nucleus (AVPV) and the periventricular nucleus (PeN), the continuum of this region known as the rostral periventricular region of the third ventricle (RP3V) (Clarkson and Herbison, 2006; Clarkson *et al.*, 2009) (Fig. 1). In contrast, humans and ruminants lack this well-defined RP3V population and have more scattered kisspeptin cell bodies within the preoptic region (Pompolo *et al.*, 2006; Oakley *et al.*, 2009; Hrabovszky *et al.*, 2010). Unlike in humans and ruminants, *Kiss1* mRNA was not detectable in the preoptic area in the adult rhesus monkey (Ramaswamy *et al.*, 2008).

Kisspeptin axons form dense pericapillary plexuses in the human infundibular stalk, the site of neurosecretion of GnRH (Hrabovszky *et al.*, 2010). Axo-somatic, axo-dendritic and axo-axonal contacts between kisspeptin and GnRH axons were also demonstrated in the infundibular stalk, in keeping with data from rodents, sheep and monkeys, where kisspeptin and GnRH neuronal networks are in close proximity (Clarkson and Herbison, 2006; Ramaswamy *et al.*, 2008; Smith *et al.*, 2008a; Hrabovszky *et al.*, 2010; Uenoyama *et al.*, 2011). GnRH neurones express *Kiss1r* mRNA (Irwig *et al.*, 2004; Han *et al.*, 2005; Messager *et al.*, 2005). These findings indicate direct involvement of kisspeptin in the neurosecretion of GnRH. However, in humans as well as other species studied to date, not all GnRH neurones receive kisspeptin neurone contacts and the incidence of these contacts seems low (Clarkson and Herbison, 2006; Ramaswamy *et al.*, 2008; Smith *et al.*, 2008a; Hrabovszky *et al.*, 2010), suggesting a subtle regulation of GnRH secretion by kisspeptin and other neuropeptides.

#### Kisspeptin neurone populations co-express other neuropeptides

There is considerable overlap in the distribution of kisspeptin, neurokinin B and dynorphin in the hypothalamus, with frequent colocalization. Mapping of kisspeptin and neurokinin B neurones was similar in the infundibular nucleus of post-menopausal women, prompting the identification of a subpopulation of kisspeptin neurones which express neurokinin B and dynorphin in the human infundibular nucleus (Rometo *et al.*, 2007; Hrabovszky *et al.*, 2010). This unique region expressing kisspeptin, neurokinin B and dynorphin is conserved across species and resides in the hypothalamic arcuate nucleus in sheep and rodents (Burke *et al.*, 2006; Goodman *et al.*, 2007; Navarro *et al.*, 2009). Neurokinin B and dynorphin are, however, absent from the kisspeptin population in the preoptic area/RP3V. These infundibular (human)E/arcuate (rodent and ruminant) nucleus neurones which co-express all three neuropeptides are referred to as KNDy neurones (Cheng *et al.*, 2010) (Fig. 1).

KNDy neurones in rats and sheep also co-localize with the glutamate transporter-2, and glutamate has been implicated in mediating estrogen positive feedback resulting in the pre-ovulatory GnRH surge. Whether KNDy cells express glutamate receptors remains to be determined (Pompolo *et al.*, 2003; Ciofi *et al.*, 2006). Kisspeptin neurones in the preoptic area/RP3V are not KNDy neurones, but in the mouse AVPV they co-express tyrosine hydroxylase (the key regulatory step in dopamine synthesis) (Oakley *et al.*, 2009). This differential expression of neuropeptides reflects complex signalling within the hypothalamus and distinct functions of the two kisspeptin populations (Ojeda *et al.*, 2010; Tello *et al.*, 2010).

#### Kisspeptin neurone populations differ in physiological function

KNDy neurones of the infundibular/arcuate nucleus influence the activity of GnRH by acting on both GnRH cell bodies and neurosecretory terminals (Krajewski *et al.*, 2005; Ciofi *et al.*, 2006; Ramaswamy *et al.*, 2008) (Fig. 1). KNDy neurones make direct contact with GnRH cell bodies and dendrites in humans and project to the median eminence in rodents, sheep and monkeys (Krajewski *et al.*, 2005; Ciofi *et al.*, 2006; Clarkson and Herbison, 2006; Ramaswamy *et al.*, 2008; Dahl *et al.*, 2009). KNDy cells act synergistically to produce coordinated and pulsatile GnRH secretion by controlling the neuroactivity of other KNDy cells, as inferred from a reciprocally interconnected KNDy cell network within the arcuate nucleus in the sheep and rat (Foradori *et al.*, 2002; Burke *et al.*, 2006; Lehman *et al.*, 2010). This is supported by the expression of neurokinin B receptors and the kappa opioid peptide receptor (the receptor for dynorphin) within the KNDy cells, but not the kisspeptin receptor, which predominantly co-localizes with GnRH neurones (Krajewski *et al.*, 2005; Navarro *et al.*, 2009; Herbison *et al.*, 2010) (Fig. 1). This implies that the stimulatory role of neurokinin B and the inhibitory action of dynorphin autosynaptically coordinate the pulsatile release of kisspeptin, which in turn drives the pulsatile secretion of GnRH and LH (Navarro *et al.*, 2009).

Kisspeptin-mediated GnRH stimulation is sex steroid dependent. Estrogen and progesterone modulate kisspeptin activity at both the AVPV nucleus and the arcuate/infundibular nucleus through sex steroid receptors (Ciofi *et al.*, 1994; Goubillon *et al.*, 2000; Foradori *et al.*, 2002; Smith *et al.*, 2005; Franceschini *et al.*, 2006) (Fig. 1). It is becoming clear that not only do kisspeptin neurones mediate both negative and positive sex steroid feedback, but also that distinct subgroups, which are species specific, are involved in these two critical regulatory functions, described more fully below (sections: Kisspeptin mediates negative sex steroid feedback and Kisspeptin may also mediate estrogenic positive feedback). In rodents, the AVPV and the arcuate nucleus respond to positive and negative sex steroid feedback, respectively (Smith *et al.*, 2005, 2006b, Herbison, 2008), whereas in humans, the infundibular nucleus alone relays sex steroid signalling (Rometo *et al.*, 2007; Oakley *et al.*, 2009) (Fig. 1). Thus while there is less marked anatomical differentiation of the two feedback pathways in humans, it remains possible (and perhaps likely) that the two functions are mediated by different neurones.

#### Kisspeptin neurones show sexual dimorphism

There is evidence for sexual dimorphism in kisspeptin pathways in the human, probably reflecting these functional differences discussed above. Female hypothalami have significantly more kisspeptin fibres in the infundibular nucleus and ventral periventricular zone than are seen in men (Hrabovszky *et al.*, 2010). There is also a striking sex difference in the number and expression of kisspeptin cell bodies, which are observed in the rostral periventricular zone of the female only (Hrabovszky *et al.*, 2010). Likewise only a few kisspeptin cell bodies are present in the infundibular nucleus in males in contrast to the abundant kisspeptin cell bodies in females (Hrabovszky *et al.*, 2010). Similarly, sex differences have been reported in the arcuate nucleus of the sheep (Cheng *et al.*, 2010). Pre-ovulatory positive sex steroid feedback is unique to the female, and the adult female mouse and rat hypothalamus contain 10-fold more kisspeptin neurones than males in the RP3V region, whereas the arcuate nucleus responsible for negative sex steroid feedback does not display such dimorphism (Clarkson and Herbison, 2006; Kauffman *et al.*, 2007).

## Kisspeptin and the regulation of GnRH secretion

Kisspeptin is a potent stimulator of the hypothalamic-pituitary-gonadal (HPG) axis in both animal models and humans. Kisspeptin signals directly to the GnRH neurones through the action on the kisspeptin receptor to release GnRH into the portal circulation, which in turn stimulates the secretion of LH and FSH from the gonadotrophs of the anterior pituitary. Evidence for this comes from multiple sources. Since GnRH cannot be measured in the peripheral circulation, LH pulse frequency remains a widely used and a well validated surrogate of hypophyseal GnRH pulsatility as each GnRH pulse is associated with an LH pulse (Clarke and Cummins, 1985).

### Kisspeptin stimulates gonadotrophin release in humans

**Figure 2:**
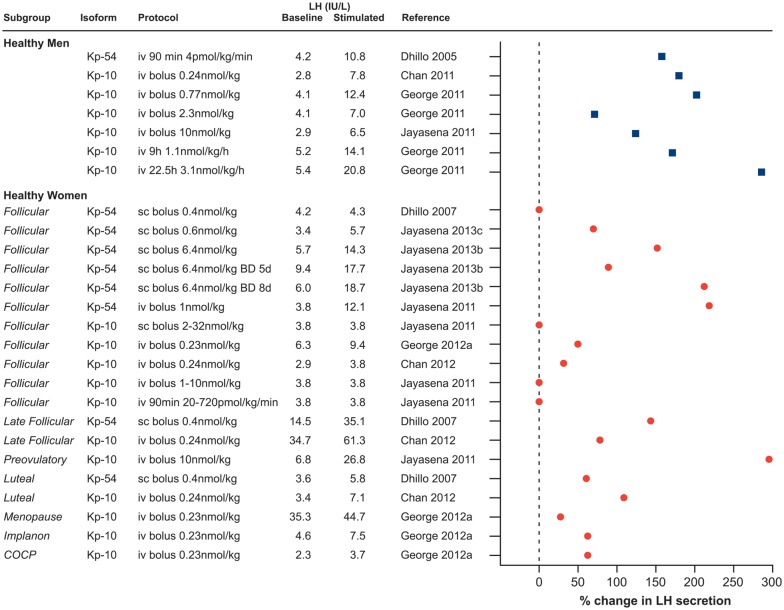
Kisspeptin stimulates LH secretion in healthy men (filled squares) and women (filled circles). The stimulatory effect of kisspeptin on LH secretion is shown in both healthy men and women, when kisspeptin is administered in different isoforms (kisspeptin-54 and kisspeptin-10) and by different protocols (intravenous or subcutaneous, single boluses or continuous infusion). Note that stimulated LH values are either mean LH or peak LH concentrations depending on how the data are originally presented. Where authors do not state exact LH concentration following kisspeptin administration, this was obtained from the relevant figures. 0% change in LH secretion is reported if no statistically significant change in LH secretion was reported and authors do not show actual LH concentrations. iv, intravenous; sc, subcutaneous; BD, twice daily; Implanon, etonogestrel contraceptive implant; COCP, combined oral contraceptive pill.

**Figure 3:**
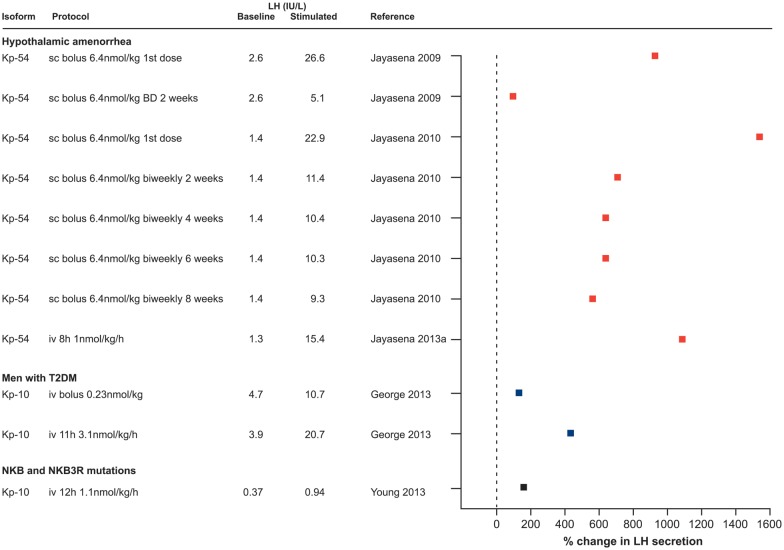
Increase in LH secretion following the administration of kisspeptin in human disease models. The stimulatory effect of kisspeptin on LH secretion is shown in reproductive endocrine disorders, when kisspeptin is administered in different isoforms (kisspeptin-54 and kisspeptin-10) and by different protocols (i.v. or s.c., single boluses or continuous infusion) as indicated. Note that stimulated LH values are either mean LH or peak LH concentrations depending on how the data are originally presented. Where authors do not state an exact LH concentration following kisspeptin administration, this is obtained from the relevant figures. BD, twice daily; T2DM, type 2 diabetes; NKB *(TAC3)*, neurokinin B; NKB3R *(TAC3R)*, neurokinin B receptor.

Kisspeptin stimulates the secretion of both LH and FSH in the human, although the effect on the former is much more marked (George and Seminara, 2012). Kisspeptin-54 was first administered in healthy men by intravenous infusion (4 pmol/kg/min (0.023 µg/kg/min) for 90 min) and resulted in a robust and dose-dependent increase (from 0.25 pmol/kg/min (0.001 µg/kg/min) to 12 pmol/kg/min (0.07 µg/kg/min)) in LH, and less marked rises in FSH and testosterone (Dhillo *et al.*, 2005). Kisspeptin-54 clearance showed first-order kinetics with a half-life of 27.6 ± 1.1 min (Dhillo *et al.*, 2005), which compares with about 4 min for kisspeptin-10 (Jayasena *et al.*, 2011). The potency of kisspeptin to stimulate the secretion of gonadotrophins and its preferential effect on the release of LH has been consistently observed when kisspeptin is administered by different routes (intravenous or subcutaneous) and types of exposure (single boluses or continuous infusion), in different isoforms (kisspeptin-54 and kisspeptin-10), to men or women or in different disease models (Dhillo *et al.*, 2005, 2007; Jayasena *et al.*, 2009, 2010, 2011, 2013a, b; Chan *et al.*, 2011, 2012; George *et al.*, 2011, 2012, 2013; George and Seminara 2012; Abbara *et al.*, 2013; Young *et al.*, 2013). Figures 2 and 3 summarize knowledge of the stimulatory effect of exogenous kisspeptin on the secretion of LH in humans to date.

While kisspeptin stimulates LH release 2- to 3-fold in most circumstances, the stimulatory effect on FSH is much smaller and is less consistent (Dhillo *et al.*, 2005, 2007; George *et al.*, 2011, 2012; Jayasena *et al.*, 2011; Chan *et al.*, 2012). A more potent effect of kisspeptin on LH secretion than FSH in humans is concordant with studies in rodents (Thompson *et al.*, 2004; Navarro *et al.*, 2005a).

### Kisspeptin operates upstream of GnRH neurones and coordinates GnRH and LH pulsatility

#### Kisspeptin acts directly on GnRH neurones

Evidence that kisspeptin exerts its stimulatory function on gonadotrophin secretion through a direct action on the hypothalamic GnRH system is provided by findings from animal studies, consistent with the anatomical studies described above. Kisspeptin causes depolarization of and increases in firing rate of GnRH neurones *in vitro* (Han *et al.*, 2005; Zhang *et al.*, 2008); kisspeptin stimulates the secretion of GnRH in hypothalamic explants (Thompson *et al.*, 2004; Tovar *et al.*, 2006); c-Fos immunoreactivity (a marker of neuronal activity) (Matsui *et al.*, 2004; Han *et al.*, 2005) and the expression of *GnRH* mRNA is up-regulated within the cell bodies of GnRH neurones following the kisspeptin exposure (Novaira *et al.*, 2009; Oakley *et al.*, 2009). In sheep, intracerebroventricular infusion of kisspeptin caused a dramatic increase in the cerebrospinal fluid GnRH content and simultaneously in serum LH and FSH (Messager *et al.*, 2005).

Some studies suggest that kisspeptin directly stimulates pituitary gonadotrophs to release LH and FSH, based on the expression of *Kiss1* and *Kiss1r* genes in gonadotrophs, the secretion of gonadotrophins from pituitary explants treated with kisspeptin (Kotani *et al.*, 2001; Navarro *et al.*, 2005b, Gutierrez-Pascual *et al.*, 2007; Richard *et al.*, 2008) and the detection of kisspeptin (although in low levels) in the hypophyseal portal circulation in the sheep (Smith *et al.*, 2008b). The ability of kisspeptin to induce gonadotrophin release from the pituitary fragments might be explained by the pharmacological concentrations of kisspeptin used (Navarro *et al.*, 2005a, Gutierrez-Pascual *et al.*, 2007). Whilst kisspeptin may have a direct stimulatory action on gonadotrophs, indirect stimulation through enhancing GnRH secretion appears to be the principal physiologic pathway for the stimulation of gonadotrophin secretion (Gottsch *et al.*, 2004; Irwig *et al.*, 2004; Smith *et al.*, 2008b).

The physiological role of kisspeptin in the regulation of GnRH secretion was further demonstrated by studies using a kisspeptin antagonist (Millar *et al.*, 2010). Kisspeptin-induced GnRH neurone firing was abolished by the kisspeptin antagonist (Irwig *et al.*, 2004; Liu *et al.*, 2008; Roseweir *et al.*, 2009). Kisspeptin is needed for the pulsatile release of GnRH, as when injected into the median eminence of pubertal rhesus monkeys, kisspeptin antagonist suppressed both mean GnRH and GnRH pulses (Roseweir *et al.*, 2009). Kisspeptin modulates the secretion of GnRH at the arcuate nucleus, the site of the GnRH pulse generator: kisspeptin antagonist reduced LH pulse frequency when administered to the arcuate nucleus but not when administered to the preoptic area in the rat (Li *et al.*, 2009).

That the effect of kisspeptin on LH release was prevented by pretreatment with GnRH antagonist further points to the action of kisspeptin upstream of GnRH (Gottsch *et al.*, 2004; Shahab *et al.*, 2005). Although there are no studies in humans administering GnRH antagonist followed by kisspeptin, the direct action of kisspeptin on GnRH neurones is inferred from consistent findings in rodents and nonhuman primates. Humans with ‘inactivating’ mutations in kisspeptin and/or its receptor show hypogonadotropic hypogonadism and delayed puberty (de Roux *et al.*, 2003; Seminara *et al.*, 2003), whist those with ‘activating’ mutations undergo precocious puberty (Teles *et al.*, 2008; Silveira *et al.*, 2010), suggesting that kisspeptin modulates GnRH pulsatility.

#### Kisspeptin increases LH pulsatility in humans

As GnRH secretion is pulsatile, the effect of kisspeptin on the characteristics of that pulsatility (as reflected in LH pulses) has been investigated. Intravenous infusion of kisspeptin-10 (1.5 µg/kg/h (1.1 nmol/kg/h) for 9 h) in healthy men (George *et al.*, 2011) and kisspeptin-54 (subcutaneous bolus 0.3 nmol/kg (1.76 µg/kg) and 0.6 nmol/kg (3.5 µg/kg)) in healthy women (Jayasena *et al.*, 2013c) increased LH pulse frequency and amplitude. The ability of kisspeptin to enhance LH pulsatility has also been demonstrated in human reproductive disorders, including in hypothalamic amenorrhoea (Jayasena *et al.*, 2013a), in hypogonadal men with type 2 diabetes (George *et al.*, 2013) and in neurokinin B signalling defects (Young *et al.*, 2013), described more fully below. Kisspeptin not only drives the pulsatile secretion of GnRH, but also appears to reset the hypothalamic clock of GnRH pulsatility in men. Acute injection of kisspeptin-10 delayed the next endogenous LH pulse by the interval that would be observed between the two consecutive endogenous LH pulses (Chan *et al.*, 2011). However, the same kisspeptin dosing protocol did not support the ability of kisspeptin to reset the GnRH pulse generator in women across the different phases of the menstrual cycle (Chan *et al.*, 2012). The authors suggest that the GnRH pulse generator in men operates differently to women and that it is the changes in the sex steroid milieu across the menstrual cycle in women that might be responsible for this discrepancy (Chan *et al.*, 2012). The marked sexual dimorphism in the anatomy of the kisspeptin system described above may underlie this intriguing observation and even determine the frequency of GnRH secretion in women across the normal menstrual cycle. This variability in the frequency of GnRH pulsatility is central to the differential regulation of LH and FSH (McNeilly *et al.*, 2003) and thus ovarian follicle development, the correct selection of a single dominant follicle for ovulation, and the luteal phase with limited follicle development.

### Men and women show sexual dimorphism in their response to kisspeptin

Men and women display sexual dimorphism in their response to exogenous kisspeptin (Fig. 2). Whilst kisspeptin potently stimulates the release of LH in men, the effect of kisspeptin is more variable in women and depends on the phase of the menstrual cycle. It has been proposed that in the early follicular phase, the impact of exogenous kisspeptin is limited due to high endogenous kisspeptin activity (Chan *et al.*, 2012), although this is speculative. Sex steroid-deficient post-menopausal women were more responsive to kisspeptin-10 than women in the early follicular phase (George *et al.*, 2012) (Fig. 2). Women taking combined estrogen and progestogen contraceptives showed a minimal response to kisspeptin-10 (George *et al.*, 2012), contrasting to the larger response in the physiological luteal phase (Dhillo *et al.*, 2007) (Fig. 2). These complex relationships suggest that other mechanisms, in addition to the absolute or relative levels of estrogen and progesterone, appear to regulate kisspeptin sensitivity across the menstrual cycle and clearly there remains much to be learnt regarding these inter-relationships. The expression of kisspeptin receptor in different sex steroid environments has not been described in primates and data in lower species remain contradictory (Navarro *et al.*, 2004a, Yamada *et al.*, 2007; Li *et al.*, 2012). Changes in pituitary sensitivity to GnRH and sex steroid feedback at that level (Hall *et al.*, 1994; Shaw *et al.*, 2010) add to the complexity of analysis of *in vivo* studies.

The sexual dimorphism in the responsiveness of men and women has been elegantly illustrated using the different isoforms of kisspeptin (Jayasena *et al.*, 2011). Men respond to modest doses of both kisspeptin-54 and kisspeptin-10. In a study of healthy women in the early follicular phase, kisspeptin-10 administered as an intravenous bolus, subcutaneous bolus or as an intravenous infusion did not result in a detectable LH response (Jayasena *et al.*, 2011) (Fig. 2). In another study however, a low-dose intravenous bolus of kisspeptin-10 induced an LH response in normal women in the early follicular phase (George *et al.*, 2012). These differences may have been methodological—as the Jayasena *et al.* (2011) protocol did not involve baseline sampling. In the study by George *et al.* (2012), a 10-min baseline LH sampling for 180 min was employed prior to kisspeptin administration, enabling comparison of LH secretion before and after kisspeptin-10 infusion in the same individual. Given the small sample number, small effect size and inter-individual variability in baseline LH, this lack of baseline data to enable intra-individual comparisons diminishes statistical sensitivity to identify small changes in LH. A small but significant increase in LH in response to kisspeptin-54 administered intravenously or subcutaneously in the early follicular phase was also observed (Dhillo *et al.*, 2007; Jayasena *et al.*, 2011), indicating that the response to the longer isoform is substantially more robust, perhaps reflecting its longer half-life. Sexual dimorphism in the kisspeptin system is also seen in rodent models: females have significantly more kisspeptin neurones than males in the AVPV nucleus (Oakley *et al.*, 2009). This sexual variation in anatomical distribution of the kisspeptin pathway and the response to kisspeptin administration may reflect sexually dimorphic roles of kisspeptin, notably in the generation of the pre-ovulatory LH surge, which is unique to the female.

### Kisspeptin mediates negative sex steroid feedback

Patterns of GnRH and subsequently LH secretion across the menstrual cycle are modulated by gonadal steroid feedback. During the follicular phase of the menstrual cycle GnRH activity and thus LH secretion is limited by estradiol (E_2_)-mediated negative feedback (with additional action on pituitary gonadotrophs) (Karsch, 1987; Shaw *et al.*, 2010). The basis for the change to positive feedback with elaboration of the mid-cycle LH surge has long been unclear. GnRH neurones do not express ERs, suggesting that a separate population of neurones acts as a mediator to relay the ovulation-inducing message from gonads to the hypothalamic GnRH neurones. Recent evidence suggests that KNDy neurones appear to constitute this ‘missing link’, mediating both negative and positive sex steroid feedback.

Kisspeptin in the infundibular nucleus mediates negative feedback of estrogen in humans (Fig. 1). In post-menopausal women kisspeptin neurones in the infundibular nucleus were hypertrophied and expressed more *KISS1* mRNA than in premenopausal women (Rometo *et al.*, 2007). These hypertrophied neurones expressed both *ESR1* (encoding ER alpha) and neurokinin B mRNA, had increased expression of neurokinin B and showed a similar distribution to that of kisspeptin neurones (Rance *et al.*, 1990; Rance and Young, 1991). The suggestion that kisspeptin and neurokinin B in the infundibular nucleus act synergistically to mediate estrogen negative feedback is supported by animal data, showing an up-regulation of *Kiss*1 mRNA expression in ovariectomised rodents, sheep and monkeys in the arcuate nucleus (equivalent to the infundibular nucleus in humans) but not in more rostral areas, and that this was prevented by E_2_ replacement (Oakley *et al.*, 2009; Lehman *et al.*, 2010). Consistent with this, the intracerebroventricular administration of kisspeptin antagonist prevented the LH rise in castrated rodents (Roseweir *et al.*, 2009). Similarly, ovariectomy increased and estrogen replacement reduced neurokinin B gene expression in the infundibular nucleus of monkeys (Abel *et al.*, 1999; Sandoval-Guzman *et al.*, 2004).

These findings together imply that estrogen mediates its negative feedback by suppressing kisspeptin and neurokinin B release from KNDy neurones, which reduces their stimulatory input to GnRH neurones (Fig. 1). The converse, i.e. inhibitory, involvement of the opioid component of this signalling system has long been recognized. The colocalization of dynorphin in at least some kisspeptin and neurokinin B-containing neurones in the human is discussed above (section: Kisspeptin neurone populations co-express other neuropeptides). Naloxone, an opioid receptor antagonist, increased serum LH levels in late follicular and luteal phases of the menstrual cycle (Quigley and Yen, 1980; Shoupe *et al.*, 1985). This effect was not apparent in post-menopausal and oophorectomized young women, whereas replacement of estrogen or progesterone restored the ability of naloxone to release LH (Melis *et al.*, 1984; Casper and Alapin-Rubillovitz, 1985; Shoupe *et al.*, 1985). The endogenous opioid peptide dynorphin mediates this role physiologically, and inhibited GnRH and LH pulse frequency following progesterone administration (Ferin *et al.*, 1984; Karsch, 1987; Goodman *et al.*, 2004). In contrast, the opioid receptor antagonist naltrexone increased serum LH concentrations and LH pulse amplitude in women with hypothalamic amenorrhoea (Genazzani *et al.*, 1995). The relative deficiency of dynorphin signalling as part of negative estrogen feedback in the post-menopausal and oophorectomized states would appear a likely explanation for the lack of response to naloxone in hypergonadotrophic states, in contrast to potentially increased dynorphin signalling contributing to the hypogonadotrophic state in hypothalamic amenorrhoea. It is however possible that already near maximal LH secretion in sex steroid deficient post-menopausal and oophorectomized women would account for the inability of naloxone to further stimulate gonadotrophin release although kisspeptin-10 induced LH secretion in post-menopausal women (George *et al.*, 2012). In post-menopausal women, the expression of prodynorphin mRNA in the infundibular nucleus is reduced (Rometo and Rance, 2008). The role of dynorphin as a mediator of sex steroid negative feedback is consistent with data from the ewe, where dynorphin is coexpressed with kisspeptin and neurokinin B, both of which show a high degree of colocalization with ER alpha and progesterone receptors in the arcuate nucleus, and the expression of prodynorphin mRNA is suppressed by ovariectomy (Goubillon *et al.*, 2000; Foradori *et al.*, 2002, 2005; Franceschini *et al.*, 2006; Goodman *et al.*, 2007). This is distinct from the apparent situation in rodents where, despite colocalization of KNDy neurones with both estrogen and progesterone receptors, dynorphin does not seem to mediate estrogen negative feedback (Navarro *et al.*, 2009).

In summary, it appears that in humans KNDy neurones mediate negative sex steroid feedback in the infundibular nucleus by suppressing the secretion of kisspeptin and neurokinin B and stimulating the secretion of dynorphin, which act synergistically to reduce the activity of the GnRH neuronal system, and thus gonadotrophin secretion.

### Kisspeptin may also mediate estrogenic positive feedback

Estrogen feedback switches from negative to positive in the late follicular phase to induce the GnRH/LH surge at the time of ovulation. However, the neuroendocrine mechanisms involved in this critical physiological event have been unclear. Emerging data suggest that although the negative feedback of sex steroids is mediated by KNDy neurones in the infundibular/arcuate nucleus, the positive feedback of sex steroids is more site and species specific (Fig. 1).

Recent data support a potential role for kisspeptin in generating the ovulatory LH surge in humans. Kisspeptin-54 (subcutaneous in doses of 1.6–12.8 nmol/kg (9.4–75 µg/kg), used instead of hCG during an FSH/GnRH antagonist assisted conception ovulation induction protocol, induced an LH surge and triggered oocyte maturation, with subsequently a live term birth reported (Abbara *et al.*, 2013). Repeated twice-daily administration of kisspeptin-54 shortened the menstrual cycle and advanced the onset of the LH peak in healthy women (Jayasena *et al.*, 2013b). This is in keeping with data from animal models. Kisspeptin administration results in an early LH surge in sheep, and conversely administration of kisspeptin antiserum or antagonists to rats and sheep prevented or blunted the LH surge (Kinoshita *et al.*, 2005; Caraty *et al.*, 2007; Clarkson *et al.*, 2008; Pineda *et al.*, 2010).

The anatomical site of kisspeptin that relays positive sex steroid feedback is different in rodents compared with humans and other species. In rodents the AVPV nucleus is the location of estrogen positive feedback, which is not matched in humans, other primates and sheep, where kisspeptin neurones in the infundibular/arcuate exert this function (Fig. 1). The expression of *Kiss1* mRNA in the AVPV nucleus is dramatically increased after estrogen replacement and at the time of the GnRH/LH surge (Smith *et al.*, 2005, 2006b). There are no studies looking at the anatomical region of kisspeptin expression that mediates estrogen positive feedback in humans and evidence comes from other species, which, like humans, have no homologous area to the AVPV nucleus. In sheep, the expression of *Kiss1* mRNA in the arcuate nucleus is markedly enhanced during the pre-ovulatory LH surge (Smith *et al.*, 2008b). In rodents the AVPV nucleus receives afferent fibres from the suprachiasmatic nucleus, the location of circadian clock, which coordinates and provides precise timing for the LH surge and the kisspeptin system in now being integrated into our understanding of the neurobiology of this system across species, including the human (Christian and Moenter, 2010; Khan and Kauffman, 2012).

KNDy neurones may have a role in positive estrogen feedback. In sheep, the neurokinin B receptor agonist senktide increased LH secretion close to levels seen during the pre-ovulatory LH surge (Billings *et al.*, 2010). KNDy neurones do not mediate positive estrogen feedback in rodents based on their location in the arcuate nucleus only (Burke *et al.*, 2006; Goodman *et al.*, 2007; Navarro *et al.*, 2009). Other neurotransmitters may also contribute to the kisspeptin-mediated LH surge, as kisspeptin populations in the preoptic area/RP3V and the infundibular/arcuate nucleus co-localize with different peptides (see above section: Kisspeptin neurone populations co-express different neuropeptides).

### Kisspeptin stimulates gonadotrophin release in disease models

In addition to being a potent stimulator of LH secretion in healthy men and women, the ability of kisspeptin to induce LH release in human disease models characterized by low gonadotrophin secretion has been investigated (Fig. 3).

#### Hypothalamic amenorrhoea

Hypothalamic amenorrhoea is characterized by slow GnRH pulsatile secretion, resulting in a preferential decline in LH compared with FSH secretion and low ovarian follicular activity. As, by definition, this is a functional rather than pathological condition, it might be readily corrected by administration of kisspeptin to increase GnRH secretion. Hypothalamic amenorrhoea was the first disease model used to explore the therapeutic potential of kisspeptin-54, which when administrated as subcutaneous bolus at 6.4 nmol/kg (37 µg/kg) resulted in a 10-fold increase in LH and 2.5-fold increase in FSH secretion, both to normal physiological levels (Jayasena *et al.*, 2009) (Fig. 3). However, this increase in gonadotrophins did not translate into a significant elevation in E_2_ secretion, suggesting that folliculogenesis was not restored, confirmed by ovarian quiescence on ultrasound scan (Jayasena *et al.*, 2009). The lack of ovarian activity may relate to the limited effect on FSH secretion and the short timescale of study. Despite the initial stimulation of LH and FSH secretion, when kisspeptin-54 was injected twice daily for 2 weeks, these increases were not sustained with LH falling to pretreatment levels, suggesting tachyphylaxis (Jayasena *et al.*, 2009, 2010) (see section: Continuous exposure to kisspeptin can cause desensitization). However, sustained secretion of gonadotrophins at physiological levels was achieved with intermittent subcutaneous injection of kisspeptin-54 twice weekly (6.4 nmol/kg (37 µg/kg)) for 8 weeks, although it did not result in increased E_2_ secretion or follicular development (Jayasena *et al.*, 2010). It has subsequently been shown that an infusion of kisspeptin-54 (0.01 nmol/kg/h (0.059 µg/kg/h) to 1 nmol/kg/h (5.9 µg/kg/h)) for 8 h in women with hypothalamic amenorrhoea can induce LH pulsatility with a 3-fold increase in LH pulse frequency and mass per pulse (Jayasena *et al.*, 2013a). The ability of the increased gonadotrophin secretion, and perhaps the relative effects of LH and FSH, to bring about ovarian activity and menstrual cycles will determine the therapeutic application of kisspeptin in this condition.

In all the studies above, regardless of the dose and route of administration, the LH response to kisspeptin is ∼5-fold greater in hypothalamic amenorrhoea than in healthy women in the early follicular phase. This is consistent with data from a rodent model of undernutrition showing up-regulated hypothalamic *Kiss1r* mRNA expression (Castellano *et al.*, 2005). Altered GnRH sensitivity is unlikely as the effect of GnRH on LH secretion is similar in hypothalamic amenorrhoea and healthy women in the early follicular phase (Jayasena *et al.*, 2009; George *et al.*, 2012).

#### Hypogonadism in men with type 2 diabetes

Men with type 2 diabetes often have low testosterone concentrations, and inappropriately low LH indicating a hypothalamic/pituitary basis (George *et al.*, 2010). As with hypothalamic amenorrhoea, increasing LH secretion by administration of kisspeptin might therefore have therapeutic potential. This has been explored in a small number of such men, investigating the response to both bolus administration and infusion of kisspeptin-10 (George *et al.*, 2013) (Fig. 3). Kisspeptin-10 intravenous bolus administration (0.3 µg/kg (0.23 nmol/kg)) increased LH secretion 2-fold in diabetic hypogonadal men, i.e. of the same magnitude as in healthy men with peak mean LH 10.7 IU/l and 14.5 IU/l, respectively (George *et al.*, 2013). An infusion of kisspeptin-10 for 11 h at a higher dose (4 µg/kg/h (3.1 nmol/kg/h)) produced a more profound 5-fold increase in LH release (George *et al.*, 2013), also comparable to the response in healthy men (George *et al.*, 2011). Kisspeptin-10 also stimulated LH pulse frequency in diabetic men with hypogonadotropic hypogonadism, which was sustained for the duration of the infusion (11 h) with no evidence of a decline in LH (i.e. no desensitization) over that timescale (George *et al.*, 2013). Importantly, serum testosterone was also elevated into the normal physiological range (George *et al.*, 2013). The ability of kisspeptin to robustly increase LH pulsatility with an associated increase in testosterone is very encouraging, but the potential of kisspeptin to maintain gonadotrophin and sex steroid release for longer periods of time relevant to therapeutic use has yet to be determined.

#### Neurokinin B signalling deficiencies

Patients with loss-of-function mutation in neurokinin B (*TAC3*) and its receptor (*TAC3R*) show hypogonadotropic hypogonadism characterized by failure to progress through puberty (Topaloglu *et al.*, 2009). It is postulated that inability of neurokinin B in an autocrine and/or paracrine manner to stimulate kisspeptin secretion results in low GnRH pulse frequency with correspondingly low LH and gonadal steroid levels but normal or near-normal levels of FSH typically seen in these patients. Neurokinin B, being potentially upstream of kisspeptin in neuroendocrine signalling, makes kisspeptin an attractive therapeutic agent to restore GnRH secretion in patients with defects in the neurokinin B system. Indeed, continuous infusion of kisspeptin-10 (1.5 µg/kg/h (1.1 nmol/kg/h) for 12 h) in two patients with *TAC3* and two patients with *TAC3R* mutation stimulated the LH response 2.5-fold (Young *et al.*, 2013) (Fig. 3). Overall, the LH response to kisspeptin was more limited than that achieved in healthy men using the same protocol (George *et al.*, 2011) with lower LH mass per pulse, although pulse frequency was normalized, consistent with complex neuropeptide interactions associated with KNDy neurone function rather than a linear hierarchy, as described above. Nevertheless, a significant increase in testosterone levels in male patients and in E_2_ levels in the single female patient was achieved (Young *et al.*, 2013).

### Continuous exposure to kisspeptin can cause desensitization

Continuing administration of GnRH desensitizes the HPG axis after an initial stimulation, by down-regulation of GnRH receptors and desensitization of gonadotrophes (Belchetz *et al.*, 1978; McArdle *et al.*, 1987; Mason *et al.*, 1994). There is evidence for pulsatile (i.e. non-continuous) secretion of kisspeptin within the hypothalamic median eminence of the monkey (Keen *et al.*, 2008). Continuous administration of kisspeptin-10 (intravenous 200 µg/h (154 nmol/kg) or 400 µg/h (307 nmol/kg) for 98 h) to rhesus monkeys resulted in suppressed LH secretion, indicative of kisspeptin receptor desensitization (Ramaswamy *et al.*, 2007). The kisspeptin receptor has also been shown to desensitize *in vitro* (Pampillo *et al.*, 2009). Consistent with this, repeated subcutaneous administration of kisspeptin-54 (6.4 nmol/kg (37 µg/kg) twice daily) for 2 weeks in women with hypothalamic amenorrhoea resulted in an initial stimulation of LH and FSH which was not maintained (Jayasena *et al.*, 2009) (Fig. 3). However other studies in humans using infusions or repeated administration of kisspeptin have not provided consistent evidence for desensitization (Figs 2 and 3). More recently, infusion of a lower dose of kisspeptin-54 for 8 h (from 0.01 nmol/kg/h (0.059 µg/kg/h) to 1 nmol/kg/h (5.9 µg/kg/h)) in women with hypothalamic amenorrhoea not only caused sustained LH secretion but also restored LH pulsatility (Jayasena *et al.*, 2013a) (Fig. 3). Continuous exposure to kisspeptin-54 administered twice daily for 1 week advanced the menstrual cycle in healthy women (Jayasena *et al.*, 2013b). Similarly, continuous kisspeptin-10 infusion at 4 µg/kg/h (3.1 nmol/kg/h) for 22.5 h in healthy men showed continuing stimulation of LH secretion, with no evidence of desensitization (George *et al.*, 2011) (Fig. 2). In contrast, LH secretion was not sustained in three healthy men during infusion of kisspeptin 10 for 24 h at 12 µg/kg/h (9.2 nmol/kg/h), the highest dose used in humans to date (Lippincott *et al.*, 2013). However, LH secretion remained well above baseline at the end of infusion, in contrast to the marked desensitization observed with a high dose of kisspeptin-54 in women with hypothalamic amenorrhoea (Jayasena *et al.*, 2009) (Fig. 2). It would be interesting to determine if LH secretion remains above baseline or if LH decreases to castrate levels with kisspeptin infusion for longer than 24 h. These data therefore suggest that while high doses of both kisspeptin-54 and kisspeptin-10 may induce desensitization, this does not occur at lower doses. In a dose-finding study involving bolus injection of kisspeptin-10, the highest dose (3 µg/kg (2.3 nmol/kg)) resulted in a sub-maximal LH response, consistent with desensitization even with bolus administration of kisspeptin-10 (George *et al.*, 2011) (Fig. 2). An alternative explanation for this observation is that at that high dose kisspeptin might have stimulated another RF-amine receptor, such as gonadotrophin inhibitory hormone receptor, known to have an inhibitory effect on GnRH and LH (George *et al.*, 2011).

Consistent with these findings, intermittent administration of kisspeptin results in sustained GnRH and LH pulsatility. Intermittent administration of kisspeptin-10 in juvenile male monkeys (intravenous hourly for 2 days) and juvenile female rats (intracerebroventricular twice daily for 5 days) caused precocious puberty (Navarro *et al.*, 2004b, Plant *et al.*, 2006). Kisspeptin-54 (6.4 nmol/kg (37 µg/kg/h)) injected twice weekly sustained the secretion of gonadotrophins for an 8-week period after a brief initial suppression (Jayasena *et al.*, 2010) (Fig. 3).

The ability of natural forms of kisspeptin to induce desensitization in humans thus remains unclear, with the discrepancies between studies possibly reflecting the duration of kisspeptin injection (8–22.5 h versus 2 weeks), lower doses of kisspeptin infused in the human studies compared with primate studies, variation in the isoforms of kisspeptin used, the mode of kisspeptin administration, differences between the human and animal models and sex and even health status (healthy men versus women with hypothalamic amenorrhoea). Kisspeptin receptor agonist analogues have also been developed, and two of these, TAK-448 and TAK-683, are potent inducers of desensitization with potential use to suppress gonadotrophin secretion and thus gonadal function, similar to the GnRH analogues widely used today. Phase I clinical studies in healthy men show that subcutaneous infusion of TAK-448 (0.01–1 mg/day) and TAK-683 (0.01–2 mg/day) for 2 weeks rapidly suppress testosterone below castration levels (MacLean *et al.*, 2013; Scott *et al.*, 2013).

## Kisspeptin and puberty

The demonstration of the obligate role of kisspeptin-GPR54 signalling in human puberty was the finding that firmly established kisspeptin as a crucial regulator of reproductive function. In 2003, two independent groups identified ‘inactivating’ point mutations and deletions in *KISS1R* that were associated with impaired pubertal development in some patients with hypogonadotropic hypogonadism (de Roux *et al.*, 2003; Seminara *et al.*, 2003). Mutations in *KISS1R* were demonstrated in both consanguineous families and in unrelated patients. In addition, Kiss1r- and Kiss1-deficient mice displayed a virtually identical phenotype (Funes *et al.*, 2003; Seminara *et al.*, 2003; d'Anglemont de Tassigny *et al.*, 2007).

Conversely an ‘activating’ mutation (Arg386Pro) in the kisspeptin receptor gene was identified in a girl with precocious puberty, although inheritance could not be determined as the biological family was not available for genetic testing (Teles *et al.*, 2008). Cells transfected with the mutant kisspeptin receptor showed prolonged accumulation of inositol phosphate and phosphorylation of extracellular signal-regulated kinase, indicating extended intracellular signalling (Teles *et al.*, 2008). Missense mutations have also been described in the *KISS1* gene in three unrelated children with central precocious puberty (Silveira *et al.*, 2010). This mutant kisspeptin is more resistant to *in vitro* degradation, suggesting greater bioavailability as the cause of precocious puberty (Silveira *et al.*, 2010).

Hypothalamic expression of *Kiss1 and Kiss1r* mRNA is dramatically up-regulated at puberty in rodents and primates (Navarro *et al.*, 2004a, Han *et al.*, 2005; Shahab *et al.*, 2005) and the percentage of GnRH neurones depolarizing in response to kisspeptin increases from juvenile (25%) to prepubertal (50%) to adult mice (>90%), suggesting that GnRH neurones acquire sensitivity to kisspeptin across puberty (Han *et al.*, 2005). Kisspeptin-54 secretion and specifically kisspeptin-54 pulse frequency increase at the onset of puberty in monkeys (Keen *et al.*, 2008). In addition to these physiological changes linking kisspeptin signalling to the timing of puberty, the administration of exogenous kisspeptin resulted in earlier puberty in rats and monkeys (Navarro *et al.*, 2004b, Plant *et al.*, 2006). Conversely, administration of a kisspeptin antagonist inhibited pulsatile GnRH release in pubertal monkeys and delayed puberty in rats (Roseweir *et al.*, 2009; Pineda *et al.*, 2010). The findings strongly support a requirement for KISS1/GPR54 signalling to initiate and progress through puberty in a range of species.

## Kisspeptin and metabolism

Human reproductive function is influenced by both extremes of nutrition—undernutrition and obesity. Kisspeptin may provide a link between nutritional/metabolic status and reproduction by sensing energy stores and translating this information into the pulsatile secretion of GnRH. The expression of *Kiss1* mRNA and gonadotrophin secretion is reduced in mice, pubertal rats and monkeys subject to fasting (Castellano *et al.*, 2005; Cota *et al.*, 2006; Roa *et al.*, 2009; Wahab *et al.*, 2011). Kisspeptin is able to restore delayed vaginal opening and increases low gonadotrophin and estrogen levels associated with chronic undernutrition in pre-pubertal rats (Navarro *et al.*, 2004b, Castellano *et al.*, 2005).

Humans with mutations in leptin or leptin receptor show hypogonadism (Farooqi and O'Rahilly, 2009). The leptin receptor (Ob-Rb) is not present on GnRH neurones, but 40% of kisspeptin neurones in the mouse arcuate nucleus express the leptin receptor (Smith *et al.*, 2006a), suggesting a role for kisspeptin in mediating the metabolic signals of leptin on the HPG axis. Leptin-deficient mice show decreased expression of *Kiss1* mRNA, which is partially up-regulated by leptin (Smith *et al.*, 2006a). Incomplete restoration of *Kiss1* mRNA expression suggests that other mediators are involved in inhibiting kisspeptin signalling in leptin deficiency. Furthermore, mice with selective deletion of leptin receptor from kisspeptin neurones display normal pubertal development, sexual maturation and fertility, demonstrating that leptin action on kisspeptin neurones is not obligatory for these processes (Donato *et al.*, 2011).

Low levels of testosterone have also been observed in men with obesity and type 2 diabetes, where decreased secretion of GnRH is thought to be the causative factor (Dandona *et al.*, 2008). A rat model of diabetes (streptozocin treated) has reduced levels of hypothalamic *Kiss1* mRNA with subsequently low levels of circulating gonadotrophins and sex steroids, which are corrected by kisspeptin (Castellano *et al.*, 2006, 2009). This raises the possibility that diminished kisspeptin secretion is a potential mechanism for hypogonadotropic hypogonadism in patients with obesity and diabetes (George *et al.*, 2010). Indeed, as described above, kisspeptin-10 increased LH pulse frequency and LH secretion in hypogonadal men with type 2 diabetes (George *et al.*, 2013). The likely pathways for down-regulation of kisspeptin signalling include negative feedback by estrogen, which is markedly elevated in obesity (Schneider *et al.*, 1979), resistance to leptin, also seen in human obesity (Finn *et al.*, 1998), insulin resistance and hyperglycaemia (Castellano *et al.*, 2006, 2009), and inflammation, which is up-regulated in hypogonadal men with diabetes (Dandona *et al.*, 2008) and is associated with decreased kisspeptin expression in rats (Iwasa *et al.*, 2008).

Current data indicate that kisspeptin acts downstream to metabolic signals and conveys information about energy stores to GnRH neurones, thereby regulating reproduction. This gives promise for a potential novel therapeutic role of kisspeptin to restore the reproductive axis in conditions of negative energy balance, such as anorexia nervosa, and in diabetes.

## Clinical applications of KNDy manipulation

**Figure 4:**
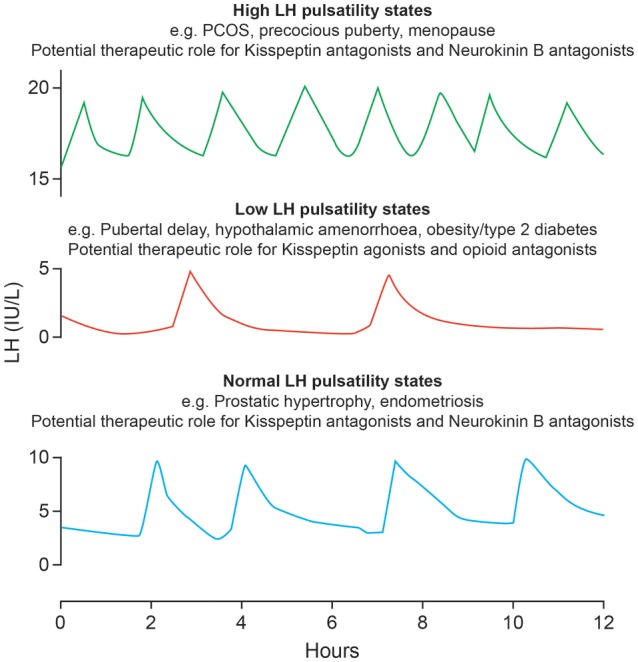
Potential clinical applications of novel kisspeptin-based modulation of LH secretion. Schematic presentation of LH pulses in health and in reproductive endocrine disorders. In health, an LH pulse occurs about every 90 min. The frequency of LH pulses is diminished in patients with hypothalamic amenorrhoea, male hypogonadism and pubertal delay, whereas LH pulse frequency is enhanced in women with polycystic ovary syndrome, menopause and precocious puberty. Therapeutic opportunities to correct abnormal LH pulse frequency by manipulating KNDy neurones with relevant agonists and antagonists are emerging. PCOS, polycystic ovary syndrome.

GnRH analogues are extensively used in clinical practice in the treatment of hormone-dependent diseases and infertility. Current therapies manipulate the HPG axis at the level of GnRH receptors on pituitary gonadotrophs, largely to suppress gonadal function, e.g. in the treatment of prostate and breast cancer, endometriosis and uterine fibroids. As reproductive endocrine conditions can be broadly categorized into those with pathologically diminished (delayed puberty, hypothalamic amenorrhoea, hypogonadism in diabetes) and pathologically enhanced (polycystic ovary syndrome (PCOS), menopause, precocious puberty) GnRH and associated gonadotrophin pulsatility, the newly discovered hypothalamic peptides kisspeptin and neurokinin B offer a novel therapeutic approach with potential advantages over the existing therapies in several clinical contexts (Fig. 4).

### Manipulation of KNDy neurones to stimulate HPG axis

Enhancing the stimulatory tone of kisspeptin and neurokinin B by appropriate agonists and suppressing the inhibitory tone of dynorphin by its antagonists, may have therapeutic potential for diseases with decreased gonadotrophin secretion. Exogenous kisspeptin enhances diminished LH pulsatility in hypogonadal men with diabetes and stimulates LH secretion in women with hypothalamic amenorrhoea (Jayasena *et al.*, 2010; George *et al.*, 2013). Kisspeptin initiates puberty in monkeys and rodents, but this has not been tested in children with delayed puberty (Navarro *et al.*, 2004b, Plant *et al.*, 2006). Pulsatile gonadotrophin secretion is restored by kisspeptin administered to patients with hypogonadotropic hypogonadism secondary to mutations in neurokinin B and/or its receptor (Young *et al.*, 2013). The role of dynorphin antagonists, such as naloxone, in patients with abnormally low LH secretion remains to be elucidated.

Kisspeptin therapy has the potential to ‘fine tune’ IVF techniques. Kisspeptin triggered the LH surge during following ovulation induction for assisted reproduction (Abbara *et al.*, 2013) with successful achievement of a live birth. Kisspeptin might stimulate a more physiological pattern of gonadotrophin secretion, avoiding the risk of ovarian hyperstimulation syndrome associated with currently used hCG injections although clearly much remains to be discovered regarding potential advantages and disadvantages over current approaches.

### Manipulation of KNDy neurones to inhibit HPG axis

Suppressing the stimulatory role of kisspeptin and neurokinin B by specific receptor antagonists and enhancing the inhibitory action of dynorphin by its receptor agonist is desirable in scenarios of increased GnRH pulsatility where a reduction rather than complete suppression of GnRH is required. Increased frequency of GnRH and therefore LH pulsatile secretion (with little effect on FSH secretion) is central to the pathophysiology of PCOS, the most common endocrinopathy in women. As GnRH pulse frequency primarily determines LH but not FSH secretion (McNeilly *et al.*, 2003), slowing GnRH might normalize the relative LH hypersecretion often seen in PCOS. Normalization of LH secretion (and perhaps the consequent hyperandrogenism) in PCOS may promote folliculogenesis and ovulation. Studies using a neurokinin B antagonist are currently underway to reduce high LH secretion in PCOS.

The ability of kisspeptin antagonists to limit follicular development and inhibit ovulation offers potential for a novel female contraceptive, perhaps being specifically advantageous in the scenarios where exogenous estrogen is contraindicated. However it might be limited by the resulting lack of progesterone exposure and adverse effects on the endometrium. Given the preferential stimulation of LH secretion in response to kisspeptin in humans (Dhillo *et al.*, 2005, 2007; George *et al.*, 2011, 2012), kisspeptin antagonists might potentially result in relative sparing of FSH compared with LH, which might reduce or prevent the unwanted side effects of estrogen deficiency, such as vasomotor symptoms and risk of osteoporosis, associated with GnRH analogue administration.

A similar therapeutic approach might support the use of kisspeptin and neurokinin B suppressive therapies in the treatment of precocious puberty. Similarly, it may alleviate menopausal hot flushes since KNDy neurones project to preoptic thermoregulatory areas that express neurokinin B receptor in rats and KNDy neurone ablation reduces cutaneous vasodilation (Burke *et al.*, 2006; Rance, 2009; Hrabovszky *et al.*, 2010; Krajewski *et al.*, 2010; Mittelman-Smith *et al.*, 2012; Rance *et al.*, 2013). Although the inhibitory role of opioids on GnRH and LH pulsatility is well known, manipulation of this system does not have the apparent specificity of the kisspeptin or neurokinin B pathways.

The potential more subtle effects of kisspeptin antagonists reducing LH pulsatility contrast with the profound suppression resulting from GnRH analogue administration, decreasing gonadotrophin and sex steroid secretion to castration levels with consequent side effects, including hot flushes, loss of libido and decreased bone mineral density (Roseweir *et al.*, 2009). Complete suppression of gonadotrophins and sex steroids is necessary is some conditions, such as prostate cancer, but partial suppression is more appropriate in benign prostatic hyperplasia, endometriosis and uterine fibroids. Clinical effectiveness in the management of endometriosis and uterine fibroids with GnRH suppression with add back, and with selective progesterone receptor modulators (Chabbert-Buffet *et al.*, 2005), suggests that approaches not based on complete suppression of the HPG axis have clear clinical value. Targeted partial gonadotrophin suppression, such as that afforded via kisspeptin and/or neurokinin B inhibition, has the potential to overcome the existing drawbacks of GnRH analogues although the emerging data on kisspeptin analogues (MacLean *et al.*, 2013; Scott *et al.*, 2013) demonstrate the potential for profound suppression as well.

## Conclusions

The discovery of kisspeptin has transformed our understanding of the neuroendocrine signals controlling the reproductive axis. Kisspeptin coordinates GnRH secretion, mediates gonadal steroid negative and positive feedback, controls the onset of puberty, and relays information regarding the body's energy stores. The last decade has thus seen a huge resurgence in interest in neuroendocrinology, and the potential for translational application is already being explored in human studies. However, much remains to be learnt before kisspeptin can replace or be used in conjunction with GnRH and gonadotrophin analogues, the current mainstay of infertility and reproductive endocrine disorder treatments.

The mode of kisspeptin administration, as with most peptides, remains a challenge and there is thus the need for novel approaches and the development of non-peptide analogues, which is already well underway. These will also allow refinement of experimental approaches to explore physiological pathways (such as elaboration of the importance of the sex steroid environment) as well as novel treatment strategies across a wide range of conditions requiring manipulation of gonadal function. Co-administration of kisspeptin, opioid and neurokinin B modifying agents will allow fine modulation of the HPG axis that may open new therapeutic avenues.

## Authors' roles

K.S., J.T.G. and R.A.A. contributed equally to determining the scope of the review. K.S. and J.T.G. undertook the literature review. K.S. drafted the manuscript, which was edited by J.T.G. and R.A.A. All authors have approved the final manuscript for submission.

## Funding

The authors' studies in this field are supported by the Medical Research Council (G0701682), the Novo Nordisk UK Research Foundation and Sanofi Excellence for Diabetes Research Awards. K.S.'s current position as a clinical research fellow is funded by the Wellcome Trust through the Scottish Translational Medicine and Therapeutics Initiative (STMTI). J.T.G.'s current position is part-funded by the University of Oxford Diabetes Trials Unit and the NIHR through the Oxford Biomedical Research Centre. Funding to pay the Open Access publication charges for this article was provided by the Wellcome Trust through the Scottish Translational Medicine and Therapeutics Initiative.

## Conflict of interest

J.T.G. serves as the International Co-ordinating Investigator for an AstraZeneca sponsored clinical trial in PCOS, as a consultant for AstraZeneca and Takeda Pharmaceuticals; and has received educational grants, speaker fees or advisory board fees from most leading pharmaceutical companies active in the field of diabetes. R.A.A. has undertaken consultancy work for AstraZeneca and Takeda Pharmaceuticals.
